# For debate: Towards standardised data collection practices for gambling helplines

**DOI:** 10.1177/14550725251341789

**Published:** 2025-06-09

**Authors:** Virve Marionneau, Søren Kristiansen, Helena Lindqvist, Inka Silvennoinen, Magnus Eidem, Lars Petter Degnepoll, Håkan Wall

**Affiliations:** Faculty of Social Sciences, Centre for Research on Addiction, Control and Governance, 3835 University of Helsinki, Helsinki, Finland; Department of Sociology and Social Work, Aalborg University, Aalborg, Denmark; Centre for Psychiatry Research, Department of Clinical Neuroscience, Karolinska Institutet, Stockholm, Sweden; Stockholm Health Care Services, 7674 Region Stockholm, Stockholm, Sweden; Peluuri, Helsinki, Finland; Department of Gambling Addiction, Blue Cross, Oslo, Norway; The Norwegian Gambling and Foundation Authority, Oslo, Norway; Centre for Psychiatry Research, Department of Clinical Neuroscience, Karolinska Institutet, Stockholm, Sweden; Stockholm Health Care Services, 7674 Region Stockholm, Stockholm, Sweden

## Introduction

Helplines are arguably the most accessible help service for gambling-related problems and harm. A recent review (Berman et al., 2023) found over 80 gambling helplines operating across the world. Helplines offer a range of low-threshold services to individuals with gambling problems and to their concerned significant others (CSOs). Services include telephone counselling, chat and email communications, referrals to other treatment, and online self-help tools such as self-tests and information. Many helplines also provide information on gambling-related issues to other health care providers (Berman et al., 2023). Helplines therefore occupy a central position in the service network for gambling-related issues, and they are usually well-known by individuals seeking help for their gambling problems (Berman et al., 2023; Clifford, 2008; Turner et al., 2022).

Alongside their help duties, most helplines collect data on customer contacts. Data are typically collected by counsellors, either by asking clients specific questions during contacts, or by filling data reports based on information that was spontaneously raised during a conversation with a client. In some cases, helplines administer client surveys either before or after contact (Berman et al., 2023). These data collection practices generate important and comprehensive datasets on gambling practices, harms and service paths experienced by individuals who have been negatively impacted by gambling. Helpline data have been described as a particularly useful data source for detecting and observing new trends in terms of harmful gambling (Wall et al., 2025).

Data collected by helplines are widely used in research. Several studies have used helpline data to assess efficacy of available interventions (Ferland et al., 2013; Rodda et al., 2014; Wall et al., 2021, 2023) or sociodemographic characteristics of help-seekers (Bastiani et al., 2015; Darbeda et al., 2020; Kim et al., 2016; Manian et al., 2024; Potenza et al., 2006; Rodda & Lubman, 2014). Helpline data have also been used to track developments in terms of products that cause harm (Marionneau et al., 2024; Rossow & Hansen, 2016; Wall et al., 2025), the impact of different policy configurations on help-seeking (Rossow & Hansen, 2016; Wall et al., 2023) and the kinds of harms that are experienced by individuals seeking help for gambling (Luquiens et al., 2021; Wall et al., 2025).

Helpline data hold a central position in tracking and monitoring gambling and gambling harms in societies, alongside other data sources such as population studies and industry data. It is therefore crucial that data collection by helplines is reliable and reflective of the reality of help-seekers and other individuals impacted by gambling harms.

The current paper outlines recent collaboration work between helplines and academics in four Nordic countries (Denmark, Finland, Norway and Sweden) aiming at standardising helpline data collection practices. This standardisation is expected to further improve gambling data infrastructures and the potential of helpline data to inform policy and practice. In the following, we present the standardisation work that has been undertaken, provide a suggested list of data collection variables for helplines and argue for key reasons why other helplines globally should also consider adopting the suggested standard for data collection.

## Collaboration to standardise helpline data collection

Already over 15 years ago, Clifford (2008) argued for a need to share and develop common minimum datasets across helplines. The need for increased collaboration across Nordic gambling helplines has also been acknowledged for many years. The Nordic region is a particularly relevant context for collaboration in the gambling harm prevention and reduction field. Nordic countries already collaborate successfully in alcohol policy and alcohol harm reduction – a field closely related to gambling (Beekman, 2023; Rehm et al., 2023). In addition, the Nordic Council of Ministries actively collaborates on various policy issues, including social and health affairs.

Nordic researchers also collaborate tightly across fields, including gambling research. For example, we have recently conducted research comparing gambling policies and consumption developments in the Nordics using helpline data (Marionneau et al., 2024; Wall et al., 2023).

As part of this collaborative Nordic gambling helpline research project, we built an initial comparative framework of helpline data variables in different Nordic countries to inform our research on which variables can be compared. Based on this initial mapping work, we found that data collection practices varied significantly across Nordic countries. Table 1 details the types of collected variables by country during the initial mapping phase, categorised into broader top-level groups.

**Table 1.** Mapping work on Nordic helpline data collection variables based on 2023 data collection lists.

Data variable	Denmark	Finland	Norway	Sweden
**All callers**				
Time and date	x	x	x	x
Duration of the call	x	x	x	x
Contact mode	x	x	x	x
Client category	x	x	x	x
**Gambling customers**				
Age	x	x	x	x
Gender	x	x	x	x
Place of residence	x	x	x	
Occupation		x		
Family status			x	
Special categories		x	x	
Medication impacting gambling		x		
***Gambling participation***				
Gambling channel	x	x	x	x
Type of gambling provider	x	x	x	
Product types causing harms*	x	x	x	x
***Consequences of gambling***				
Duration of gambling problems	x	x		x
Problem gambling screener	x			
Crime-related harms		x	x	x
Suicidality	x	x	x	x
Health harms	x	x	x	x
Relationship harms		x	x	
Harms to children		x	x	x
Work harms		x	x	
Gambling-related debt	x	x	x	
Co-morbidities	x	x		
Motivations for gambling		x		
***Help-seeking***				
Previous help-seeking	x	x		x
Sought help			x	x
Treatment or help referrals	x	x		x
**CSO callers**				
CSO background information	x	x		x
Background information on gambling individual	x	x		x
CSO relationship to gambling individual	x	x		x
Information on harmful gambling	x	x		
CSO reasons for contacting helpline				x
Harms experienced by CSO		x		
Help referrals for CSOs	x	x		x
**Professional callers**				
Type of professional	x	x		x
Type of contact	x	x		
**Total number of variable types**	25	33	20	22

*A comparative list of gambling types causing harm was developed for and is available in Marionneau et al. (2024).

CSO = concerned significant other.

Based on this initial data list, we conducted two online workshops between the research team and the Nordic gambling helpline representatives during 2024 to discuss ways forward for standardising the variable list. After the first workshop, we produced an initial proposition for a standardised list. This list was then discussed further in a second workshop, producing a formalised final version. The recommended variables for gambling customers are provided in Table 2. Recommended variables for CSO customers are provided in Table 3.

**Table 2.** Recommended standardised variables for gambling customers at helplines.

Variable	Answer options
**Background variables**	
Age (years)	Under 18 / 18–24 / 25–34 / 35–44 / 45–54 / 55–64 / 65–74 / over 74
Gender	Female / male / other
County / Region	Country-specific categorisations
How long has gambling been a problem for you?	Open-ended
Is the customer a repeat caller	Yes / No / Unknown
– If yes, is the customer regular caller?	Yes / No
– If yes, was the last contact within the ongoing year?	Yes / No
**Gambling behaviours**	
Which gambling formats cause problems or harms for you	EGMs online EGMs land-based Casino online Casino land-based Casino products in restaurants Poker online Poker land-based Sports betting online Sports betting land-based Horse betting / toto online Horse betting / toto land-based E-sports betting Crash games Bingo online Bingo land-based Lotteries online Lotteries land-based Scratch cards online Scratch cards land-based Gambling or trading with cryptocurrencies Skin betting Gambling-like features in video games (loot boxes) Stock market trading Other (with empty box to fill in what)
**Channelling**	
Are you self-excluded from licensed gambling but continue gambling via other channels?	Yes / No
Do you gamble on licensed offer or offshore offer?	Licensed gambling only / mostly / Offshore gambling only / mostly / both channels equally / I don't know
**Harms**	
Have you or anyone close to you experienced financial problems due to your gambling?	No / Yes, but not in the past year / Yes, in the past year
Has your gambling worsened your mental health?	No / Yes, but not in the past year / Yes, in the past year
Have you experienced serious problems in any important relationship because of your gambling?	No / Yes, but not in the past year / Yes, in the past year
Have you experienced serious problems at work or in school because of your gambling?	No / Yes, but not in the past year / Yes, in the past year
Do you ever think that you would be better off dead, or that you would hurt yourself due to gambling?	No / Yes, but not in the past year / Yes, in the past year
Do you have debts due to gambling?	Yes / No
– If yes, how large are your gambling-related debts?	Under 1000 euros 1001–5000 euros 5001–10,000 euros 10,001–20,000 euros 20,001–50,000 euros 50,001–100,000 euros over 100,000 euros
– If yes, to whom are you indebted to:	Banks / Instant loan providers / private individuals / Other, what [empty box]

EGM = electronic gaming machine.

**Table 3.** Recommended standardised variables for CSO customers at helplines.

Variable	Answer options
**Background variables**	
Age (years)	Under 18 / 18–24 / 25–34 / 35–44 / 45–54 / 55–64 / 65–74 / over 74
Gender	Female / male / other
County / Region	Country-specific categorisations
How long has gambling been a problem for you?	Open-ended
Is the customer a repeat caller	Yes / No / Unknown
– If yes, is the customer regular caller?	Yes / No
– If yes, was the last contact within the ongoing year?	Yes / No
Relationship to gambling individual	Partner / Ex-partner / Mother / Father / Sibling / Child / Other relative / Friend / Colleague / Employer / Other [empty box]
How long has gambling been a problem for your close one?	Open-ended
**Harms**	
Have you or anyone close to you experienced financial problems due to someone else's gambling?	No / Yes, but not in the past year / Yes, in the past year
Has someone else's gambling worsened your mental health?	No / Yes, but not in the past year / Yes, in the past year
Have you experienced serious problems in any important relationship because of someone else's gambling?	No / Yes, but not in the past year / Yes, in the past year
Have you experienced serious problems at work or in school because of someone else's gambling?	No / Yes, but not in the past year / Yes, in the past year
Are you impacted by gambling-related debt due to someone else's gambling?	No / Yes, but not in the past year / Yes, in the past year / I don’t know
Do you ever think that you would be better off dead, or that you would hurt yourself due to someone else's gambling?	No / Yes, but not in the past year / Yes, in the past year
Do you experience or fear emotional or physical abuse due to someone else's gambling?	No / Yes, but not in the past year / Yes, in the past year
**Optional questions**	
Do you know which gambling products are causing problems or harms to the gambling individual?	[Same categories as in Table 2 + I don’t know]
Do you know if the gambling individual is self-excluded from licensed gambling but continues gambling via other channels?	[Same categories as in Table 2 + I don’t know]
Do you know if the gambling individual primarily gambles on licensed offer or offshore offer	[Same categories as in Table 2 + I don’t know]

CSO = concerned significant other.

Most variables in Tables 2 and 3 are adapted from the most comprehensive existing data collection practices of individual helplines. For some background variables, notably age and county/region, we opted for top-level categories instead of open-ended answers to protect the identity of customers. Harm items for both sets of questions are based on the harm questions of the Gambling Disorder Identification Test (GDIT) (Molander et al., 2023). The suicidality question is adapted from a suicidality question in the Patient Health Questionnaire (PHQ-9) survey for depression screening.

Based on discussions in our workshops, we omitted questions related to gambling habits and debt levels from the recommended CSO questionnaire because most helpline representatives felt that CSO callers rarely have detailed information about the gambling habits of their close one. Furthermore, it is in the interest of helplines to focus primarily on the wellbeing of CSOs if they are the customer. Questions related to harmful gambling products and channelling were nevertheless included as optional questions for CSOs that helplines can include based on their own judgement.

The questions presented in Tables 2 and 3 were designed to be asked during all contacts, if possible. All helplines are also welcome to include additional country and context-specific questions. Helplines have also introduced additional “did not answer” or “left before answering” options to all the questions.

## Implementation of the standardised questionnaire

National gambling helplines in Denmark, Finland, Norway and Sweden have implemented most or all of these questions in their data collection as of January 2025. Some of the helplines omitted individual questions or answer options in our standardised questionnaire due to national practices. For example, the Swedish helpline does not screen for suicidality, while the Finnish helpline did not implement the question on self-exclusion. Countries also differ to some extent in terms of available gambling products. Most questions were nevertheless agreed to by all helplines.

The first impressions of the questions were discussed in a third meeting in March 2025. Going forward, the team plans to meet regularly to exchange experiences and observations on the data collection and to further improve the questionnaire and its compatibility with help work, including motivational interviewing.

Based on first impressions, the new variable list has been well-received amongst helpline counsellors. Despite some initial reservations and the need for familiarisation, helpline counsellors have reported that most questions are answered naturally during contacts. Questions are asked either in the original wording or as part of the conversation. Questions that are not answered during contacts are usually asked at the end of the conversation. Particularly background questions can sometimes be difficult to ask naturally. For chat conversations, helplines are implementing some of the variables as pre-questions that customers can answer while waiting in the queue for a counsellor to respond.

Counsellors report that the standardised questionnaire has provided structure to conversations and the questions have been helpful. While the questionnaire cannot be filled for all contacts, the quality of data collection has also improved compared to the previous situation. Helplines have not had feedback on the standardised questionnaire from customers, suggesting that the questions are not considered invasive or annoying by those to whom they are addressed.

## Benefits of adopting standardised data collection practices at gambling helplines

We hope that our work to standardise data collection practices at Nordic gambling helplines can serve as an example to other helplines internationally. Helplines operate in over 80 countries and have a wide reach with individuals experiencing gambling harms (Berman et al., 2023). Helplines have an important role in providing treatment and help, but they can also contribute to driving the evidence base of the negative impacts of gambling globally. We can identify three key benefits in producing comparable and standardised helpline data also beyond the Nordic context.

First, standardised data collection and collaboration across helplines can inform better targeting of interventions and improve the reach of help services. Helpline services can reduce gambling and gambling-related problems (Sciola et al., 2024), but only a minority of individuals experiencing gambling problems seek any help (Bijker et al., 2022). Comparative evidence of how and why individuals seek help, which specific types of services they prefer and what kind of harms they experience, can be mobilised to increase awareness and accessibility of helplines and to target services to differing customer needs.

Second, better comparable data strengthens research and data infrastructures. Helpline data are important in informing research and policy on help-seeking, gambling harms and impacts of different regulatory practices. Helpline data are complimentary to other data sources, such as population studies, in that they provide access to larger groups of individuals experiencing gambling harms (Berman et al., 2023). Helpline data can have a lesser response bias than data from population surveys: Helpline customers are more likely to be honest about their experiences to receive appropriate help (cf. Goldstein et al., 2017). Helpline data are collected regularly in day-to-day work. They can therefore be flexible in identifying new and emerging patterns in gambling harms. It is important that researchers continue to have access to high-quality data from helplines to produce best possible evidence on the public health impacts of gambling.

Third, standardisation can help promote increased international collaboration in the gambling field. International collaboration and standards for reducing harms are already prevalent in fields such as tobacco control and alcohol policy (e.g., World Health Organization (WHO) Framework Convention on Tobacco Control; WHO Best Buys for alcohol policy). Similar international collaboration is urgently needed in gambling harm prevention and reduction. Commercial gambling is widely accessible and rapidly growing across the world (Ukhova et al., 2024). The expansion of gambling, particularly online, is causing significant harm to individuals and populations (Tran et al., 2024). Addressing harms generated by this increasingly global and digital industry is no longer possible with jurisdiction-specific control structures (Wardle et al., 2024). Better data – including from helplines – can help highlight the harms of gambling at a global level and push for more stringent international collaboration and standards in gambling harm prevention and policy.

For these reasons, we strongly encourage other helplines globally to follow the example of the Nordic countries and to adopt a more standardised template for their data collection. The standardised Nordic surveys for gambling individuals and CSOs (Tables 2 and 3) can serve as examples for these developments.

## Conclusions

We have outlined work conducted in the Nordic countries to standardise helpline data collection. Standardised helpline data is an important piece in the puzzle of effective gambling harm monitoring. With comparable data, we can better understand the global ramifications of commercial gambling and its effects. Standardised helpline data collection can help us compare developments across societies, generate research evidence on the impacts of different regulatory configurations, and enable further development of help services. International collaboration and better data are keys to achieving these goals. Helplines can provide crucial information to accomplish better monitoring and understanding of the harmful and emerging impacts of gambling on public health.
